# Introduction of Subcutaneous Depot Medroxyprogesterone Acetate (DMPA-SC) Injectable Contraception at Facility and Community Levels: Pilot Results From 4 Districts of Uganda

**DOI:** 10.9745/GHSP-D-18-00117

**Published:** 2018-12-27

**Authors:** George Odwe, Kate Gray, Annet Kyarimpa, Francis Obare, Grace Nagendi

**Affiliations:** a Population Council, Nairobi, Kenya.; b International Planned Parenthood Federation, London, United Kingdom.; c Reproductive Health Uganda, Kampala, Uganda.; d International Planned Parenthood Federation, Africa Region, Nairobi, Kenya.

## Abstract

Reproductive Health Uganda (RHU), a local NGO, introduced subcutaneous depot medroxyprogesterone acetate (DMPA-SC, brand name Sayana Press) in 4 districts of Uganda between April 2016 and March 2017. RHU trained public and private facility providers on all family planning methods including DMPA-SC; trained community health workers (known as village health teams, VHTs) to give family planning counseling, provide short-acting methods including DMPA-SC, and make referrals for long-acting and permanent methods; conducted mobile outreach and raised awareness of family planning; and provided family planning commodities. We used a retrospective cross-sectional evaluation design drawing on data from (1) in-depth interviews with 32 facility- and community-based providers; (2) key informant interviews with 7 policy makers and program staff; and (3) family planning program statistics from 4 RHU clinics, 26 mobile outreach sites, and 40 VHTs in 4 study districts. Data collection took place between April and June 2017. Over 12 months, 14,273 units of DMPA-SC were provided in RHU clinics, by mobile outreach teams, and by VHTs. DMPA-SC units were mostly administered in community settings either by VHTs (70%) or at mobile outreach events (26%). A substantial proportion (43%) of DMPA-SC units were administered to young people (<25 years), a significantly higher proportion compared with other methods provided to this age group through the project (*P*<.001), except condoms. In addition, a greater proportion of DMPA-SC units provided at the community level by VHTs were used by young people (45%) compared with units provided at outreach (36%) or in clinics (35%). Overall, injectables (DMPA-SC and intramuscular DMPA combined) came to represent 43% of all contraceptive methods provided, up from a baseline of 20%. This shift occurred despite significant increases in the volume of all other methods provided (*P*<.001). Qualitative data revealed various factors that facilitated introduction, including comprehensive training, commodity availability, strong referral links, and early community engagement. RHU's experience supports the viability of community-based delivery of DMPA-SC and identifies opportunities to strengthen this approach. There is further evidence that DMPA-SC may be popular with young people, especially in community settings.

## Abstract (precis)

Over 1 year, the NGO-led project provided more than 14,000 units of DMPA-SC, mostly in community settings and to a substantial proportion (43%) of young women. The share of injectables increased significantly, as did the volume of all methods provided, including short-acting, long-acting, and permanent methods.

## INTRODUCTION

An estimated 214 million women and girls in low- and middle-income countries (LMICs) would like to stop having children or delay their next birth for at least 2 years but are not using a modern contraceptive method and are therefore at risk of unintended pregnancy.^1^ Limited access to family planning services and restricted method choice have been cited as main reasons for high levels of unmet need for contraception in LMICs.^2^ In an effort to increase the availability of a range of contraceptive methods, and in doing so, potentially attract new voluntary family planning users, a new injectable contraceptive known as subcutaneous depot medroxyprogesterone acetate (DMPA-SC) has been developed.^3^

DMPA-SC is a 3-month, progestin-only injectable contraceptive containing 104 mg of DMPA per 0.65 ml dose that is administered into certain fatty areas under the skin. Sayana Press (a brand of DMPA-SC developed by Pfizer) combines the drug and needle in a single prefilled Uniject system (a trademark of Becton, Dickinson and Company), designed only for single use. The single-unit design makes DMPA-SC easy to transport and simple to administer as providers do not have to draw a measured dose into the syringe from a vial.^4^^,^^5^ These features make it suitable for community-based distribution and for women to administer themselves, thus avoiding the need for them to travel to a health facility.^6^ In 2018, the World Health Organization (WHO) included DMPA-SC in its revised *Essential Medicines List*.^7^ DMPA-SC is currently approved for use in a number of sub-Saharan African countries including Burkina Faso, Niger, Senegal, and Uganda.^8^

The single-unit design of Sayana Press makes it easy to transport and simple to administer.

To date, the bulk of evidence on DMPA-SC has come from high-quality research trials on the method's acceptability, efficacy, and safety. However, there is still much to learn about different models and approaches for DMPA-SC rollout and scale up. In 2016/2017, Reproductive Health Uganda (RHU)—a member association of the International Planned Parenthood Federation—implemented a 1-year project to increase learning on provision of DMPA-SC at the facility and community levels. The project, also known as the Sayana Press Learning Project, was implemented in 4 districts of Uganda—Kabale, Kabarole, Mbale, and Mbarara.

Estimates from the 2016 Uganda Demographic Health Survey show that about 41% of pregnancies in Uganda are unintended.^9^ Teenage pregnancy is also highly prevalent—approximately 25% of adolescent girls aged 15–19 in Uganda have begun childbearing.^9^ However, the modern contraceptive prevalence rate (mCPR) is modest at 35% among married women aged 15–49 years.^9^ The Ugandan government has made commitments to the Family Planning 2020 (FP2020) initiative to improve access to family planning services, with the goal of reducing unmet need for family planning from 38% to 10% and increasing mCPR to 50% by 2020.^10^ To help achieve these targets, Uganda is implementing a community-based health strategy allowing community health workers, known as village health teams (VHTs) in the country, to provide both family planning information and selected services including injectable contraceptives and other short-acting methods (condoms and oral pills) in their community.^11^^–^^15^ In 2014, the Ugandan government launched a pilot introduction of DMPA-SC through the VHT program across 28 districts to accelerate achievement of FP2020 goals. RHU was one of a range of partners involved in this initial pilot introduction of DMPA-SC and served as the first private-sector NGO clinic entry point for the DMPA-SC pilot in Gulu district, northern Uganda.

In this article, we document RHU's experience in introducing DMPA-SC services in 4 other districts of Uganda through various models, consisting of static clinics, community-based distribution through VHTs, and mobile outreach in remote communities by a team of trained providers. We also share lessons learned from RHU's experience in supporting the rollout of DMPA-SC from the perspective of key informants involved in the project including service providers, project staff, and government officials. The findings can inform policy makers and program implementers in other countries on how to introduce DMPA-SC into the range of available methods.

## PROJECT DESCRIPTION

### Study Setting

RHU is a Ugandan NGO with a network of 17 clinics across the country that offer integrated sexual and reproductive health information and services including comprehensive family planning, testing and treatment for HIV and other sexually transmitted infections, and cervical cancer screening. RHU provides these integrated services through clinic-based providers, mobile outreach teams, and community resource persons, including VHTs. RHU implemented the Sayana Press Learning Project from April 2016 through March 2017 in collaboration with the Ministry of Health (MOH), mainly in partnership with public-sector facilities (health centers) in remote settings. In selecting project districts, RHU prioritized (1) districts with well-established RHU clinics offering a full range of voluntary family planning services and capacity to support local public-sector facilities and community health workers during the pilot, and (2) districts where no other DMPA-SC implementation had taken place through other pilots. Table 1 presents current reproductive health indicators for the sub regions where study districts are located.

**TABLE 1.** Reproductive Health Indicators for Study Districts, Uganda, 2016

District	Subregion	TFR	Teenage Pregnancy (%)	mCPR (%)	Unmet Need for Family Planning (%)
Mbale	Bugisu	5.6	28.2	43.2	22.1
Kabarole	Toro	5.4	30.3	37.4	41.3
Mbarara	Ankole	4.9	18.9	36.2	44.5
Kabale	Kigezo	4.6	15.5	43.2	NA
**National**		**5.4**	**24.8**	**34.8**	**31.9**

Abbreviations: NA, not available (due to small numbers of data); mCPR, modern contraceptive prevalence rate; TFR, total fertility rate.

Source: UBOS and ICF (2018).^9^

Table 2 lists programmatic inputs. In order to achieve integration of DMPA-SC into the family planning service delivery structure at the community level and strengthen family planning services in static clinics, RHU provided support in 5 key areas: training; demand creation; commodity supply; partnerships and collaboration; and service delivery at community and facility levels.

**TABLE 2.** Sayana Press Learning Project Activities by Service Delivery Channel, Uganda, April 2016–March 2017

	Service Delivery Channels			
	VHTs	Static Clinic		Mobile Outreach
		Public Sector	RHU Clinics	
**Number supported**	40 VHTs (40% men, 60% women; 10 VHTs per district selected from existing cadre of active VHTs)	26 clinics with outreach site Kabale (9) Kabarole (4) Mbale (6) Mbarara (7)	4 clinics (1 RHU static clinic per project district)	Mobile outreach conducted in 26 public-sector clinic sites
**Service delivery**	Comprehensive family planning counseling, information, and awareness raising Short-acting methods (DMPA-IM, DMPA-SC, oral contraceptives, condoms) Referral to mobile outreach teams, RHU clinics, or public-sector clinics for long-acting methods and other SRH services	Comprehensive family planning counseling and information Short-acting methods (DMPA-IM, DMPA-SC, oral contraceptives, condoms) Long-acting methods (implants and IUDs) Permanent methods (tubal ligation and vasectomy) Other SRH services (including HIV and STI testing/treatment and cervical cancer screening)		
**Trainings**	All 40 VHTs received a 7-day training on: Comprehensive counseling on all methods (including DMPA-SC) Provision of DMPA-SC Provision of other short-acting methods Referral processes Project data collection	42 facility-based providers (nurses, midwives, and clinical officers) drawn from public-sector facilities with outreach site and RHU facilities received a 2-day training on: Comprehensive counseling on all methods including DMPA-SC Provision of DMPA-SC Refresher training on all family planning methods		
**FP supplies and equipment**	VHTs obtained FP commodities from RHU clinics in project districts	FP commodities provided through the NMS	All FP commodities (including DMPA-SC) provided through UHMG	All FP commodities and equipment provided by RHU outreach teams
**Additional support provided**	Monthly supportive supervision provided by RHU clinical staff Comprehensive counseling on all methods including DMPA-SC	NA	NA	NA
**Demand creation**	1 stakeholders' meeting involving 118 participants (including district health officers and religious and community leaders) drawn from all 4 project districts Educational outreach by RHU providers or VHTs about importance of family planning method through door-to-door mobilization, group information sessions, IEC materials, and media (mainly TV and radio programs)			

Abbreviations: DMPA-IM, intramuscular depot medroxyprogesterone acetate; DMPA-SC, subcutaneous depot medroxyprogesterone acetate; FP, family planning; IEC, information, education, and communication; IUD, intrauterine device; NA, not applicable; NMS, National Medical Stores; RHU, Reproductive Health Uganda; SRH, sexual and reproductive health; STI, sexually transmitted infection; UHMG, Uganda Health Marketing Group; VHT, village health team.

RHU provided support in training, demand creation, commodity supply, and service delivery in order to integrate DMPA-SC into family planning services at the community and facility levels.

### Training

A total of 42 facility-based providers, mainly nurses, midwives, and clinical officers, primarily from public-sector facilities with outreach sites as well as from RHU's own facilities, were trained on DMPA-SC using modules developed by PATH. Public-sector providers also received refresher training on all family planning methods to be able to support mobile outreach services in their respective facility sites. In addition, RHU recruited 40 active VHTs (10 in each study district) and trained them for 7 days using the MOH's national curriculum for VHTs and DMPA-SC training modules developed by PATH. The VHTs' training was aimed at improving their knowledge, skills, and competencies on counseling family planning clients on all methods; provision of short-acting methods including DMPA-SC; community mobilization; reporting; and referrals. To ensure service quality, RHU clinical staff conducted monthly supportive supervision meetings with VHTs participating in the project. At these meetings, existing MOH tools were used to assess and strengthen VHT skills.

### Demand Creation

RHU supported VHTs to mobilize communities to attend outreach events and to provide information about family planning when visiting households in their communities. In addition, RHU providers conducted family planning health education sessions at health centers during outreach visits. The project also used the media (mainly radio talk shows), billboards, and community dialogue sessions to increase access to information about family planning, including DMPA-SC.

To update community stakeholders about the family planning services available through the project and to ensure community acceptance of planned activities, the project team held meetings with community leaders from project districts prior to implementation. Information about DMPA-SC was shared with participants during these stakeholder workshops.

### Commodity Supply

In line with standard supply-chain processes in Uganda, public health facilities in project districts received commodities from the Ugandan National Medical Stores. Like all NGO partners that collaborated with the MOH to deliver family planning services in Uganda, RHU received contraceptive commodities including DMPA-SC via the Uganda Health Marketing Group (UHMG) and distributed them to all its service delivery points in the participating districts.

### Partnerships and Collaboration

RHU was a member of the DMPA-SC coordination group that spearheaded the rollout of DMPA-SC services nationally. Members of the DMPA-SC coordination group met on a monthly basis to track DMPA-SC introduction progress, identify and respond to emerging challenges, and make decisions about national DMPA-SC introduction. The coordination group included representatives from the MOH and other NGOs implementing DMPA-SC rollout activities in other districts. Members of the coordination group complemented each other to support DPMA-SC activities. For example, RHU adopted the DPMA-SC educational and training materials developed by PATH to train providers.

### Service Delivery

RHU implemented the Sayana Press Learning Project within the existing family planning service delivery structures, via 3 principal channels: VHTs, static clinics, and mobile outreach. **At the community level,** the project supported integration of DMPA-SC into family planning service delivery via:

- **40 VHTs (10 from each district)**, who provided comprehensive family planning counseling; delivered DMPA-SC along with other short-acting methods to women in their homes and in community settings; and referred clients interested in long-acting methods to mobile health units, RHU clinics, or public-sector static clinics. To enable them to travel within their communities and to clinics to restock family planning commodities, VHTs received a monthly transportation allowance of approximately 30,000 Ugandan shillings (about US$8 at 2018 exchange rates). In setting this allowance, the project used standard RHU rates for 2015/2016, which were lower than the rate paid by comparable NGOs but higher than the government rate of US$3 per month.
- **Mobile outreach teams,** who provided a mix of long- and short-acting family planning methods (including DMPA-SC). Each team consisted of 1 driver, at least 1 VHT to mobilize communities, and a mix of RHU outreach clinical staff and project-trained public-sector service providers—mainly nurses and midwives.

DMPA-SC was provided through 3 principal channels: village health teams, static clinics, and mobile outreach.

**At the facility level,** providers at RHU and government static clinics provided all family planning services including long-acting methods and DMPA-SC as a new method.

## METHODS

We used a retrospective cross-sectional evaluation design, drawing on both qualitative and quantitative data. Data collection took place from April to June 2017 in the 4 project districts.

Qualitative data were collected through in-depth interviews with 20 VHTs (5 per project district) and 12 facility-based health workers (4 from RHU clinics and 8 from public-sector facilities) to assess their experiences and their perceptions of enablers of and barriers to DMPA-SC service provision. Only providers who participated in the program were eligible for inclusion. In addition, we conducted 7 key informant interviews (2 national and 4 district-level policy makers and 1 program staff) to assess how the project was implemented and to seek their views on the feasibility of scaling up DMPA-SC services. Key informants were selected based on their role in the intervention. Written informed consent was obtained from all participants before conducting the interviews. Interviews were audio-recorded, transcribed, and translated into English when necessary.

Quantitative data were based on family planning service statistics extracted from RHU's static clinics, VHTs, and mobile outreach activities in the study districts. For each service delivery channel (i.e., static clinics, VHTs, and outreaches), information was collected on the number of family planning services provided by age category of recipients (below 25 years and 25 years or older). Family planning service data were captured using standard registers, which had been adapted to include DMPA-SC as a new family planning method. At the community level, VHTs captured family planning client records using VHT registers, which were submitted to RHU facilities on a monthly basis. Prior to the start of the project, RHU trained VHTs and RHU's facility-based providers on data collection, storage, and reporting procedures. Data from each service delivery unit were entered into RHU's District Health Information Software (DHIS), which is a centralized platform. Information Management Assistants based at RHU clinics in each project district were responsible for data management and quality assurance across all service delivery points. In addition, monthly data quality assessment visits to RHU facilities were conducted by headquarters staff. We did not capture family planning data from the public health facilities in project sites as DMPA-SC had not been integrated into the MOH management information system by the time of this evaluation.

We downloaded family planning data into Microsoft Excel, and then exported the data to Stata version 11 for analysis. Quantitative data analysis involved generating descriptive statistics, mainly via cross-tabulations and frequencies, and conducting test on proportions of family planning services to examine whether there were any significant changes between the 6-month period prior to project implementation and the second 6-month period of project implementation (i.e., months 7 through 12 of project implementation). We used Impact 2 (version 5) to calculate couple-year of protection (CYP) estimates.^16^ We analyzed qualitative data using content analysis by coding the data and identifying common themes based on the interview guides.

### Ethical Clearance

Ethical approval for the study was obtained from the Population Council's Institutional Review Board (Protocol 762) and The AIDS Support Organization (TASO) Research Ethics Committee in Uganda (TASOREC/38/16-UG-REC-009). The Uganda National Council for Science and Technology (UNCST) granted administrative permission for the research (SS4225).

## RESULTS

### Volume of DMPA-SC Units Provided

A total of 14,273 units of DMPA-SC were distributed by VHTs, at RHU clinics, and through mobile outreach in project districts between April 2016 and March 2017 (Table 3). The mean number of units administered across all project sites and through all service delivery points (excluding public-sector static clinics) was 1,189 units per month, with a high of 1,757 units distributed in March 2017 and a low of 200 units during the first month of implementation (April 2016).

More than 14,000 units of DMPA-SC were distributed over the 12-month project period.

**TABLE 3.** Number of DMPA-SC Units Provided by Project District and Service Delivery Channel, Uganda, April 2016–March 2017

District	Service Delivery Channel			Monthly Average No.	Total No.
	Clinics No. (%)	Outreach No. (%)	VHTs No. (%)		
Mbale	131 (2.7)	1060 (22.2)	3589 (75.1)	4780	398
Kabarole	110 (3.5)	881 (28.0)	2157 (68.5)	3148	262
Mbarara	87 (4.2)	405 (19.6)	1579 (76.2)	2071	173
Kabale	267 (6.2)	1388 (32.5)	2619 (61.3)	4274	356
**Total**	**595 (4.2)**	**3734 (26.2)**	**9944 (69.7)**	**14,273**	**1189**

Abbreviations: DMPA-SC, subcutaneous depot medroxyprogesterone acetate; VHT, village health team.

**FIGURE 1.**
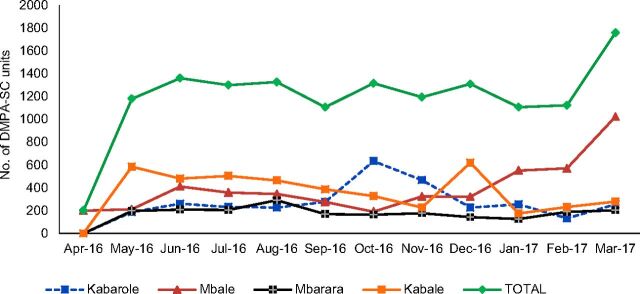
Monthly Trends in the Number of DMPA-SC Units Provided by District, Uganda, April 2016–March 2017 Abbreviation: DMPA-SC, subcutaneous depot medroxyprogesterone acetate.

There were some fluctuations in the monthly distribution of DMPA-SC during the project period (Figure 1). These fluctuations could be attributed mainly to program challenges such as delays by VHTs in replenishing their family planning stocks and delays in disbursal of funds to RHU and implementation teams.

### Volume of Family Planning Methods Provided

Table 4 presents the number of all family planning methods delivered during the 12-month project period and for 6 months before the project began. A total of 82,254 family planning methods, representing approximately 24,500 CYPs, were delivered during the 12 months of project implementation. This translates to a mean of 6,854 units (2,042 CYPs) per month, up from a mean of 3,570 units (1,179 CYPs) per month during the 6 months prior to project implementation, representing a substantial growth in the volume of methods provided across all sites. There were also notable and significant increases in the volume of each individual method provided through the project, including condoms (male and female), oral contraceptives, contraceptive implants, intrauterine devices (IUDs), and permanent methods (*P*<.001 for all methods).

There were significant increases in the volume of all family planning methods provided through the project.

**TABLE 4.** Volume of Family Planning Methods Provided Before and During the Intervention by Contraceptive Method, 4 Districts of Uganda

Method	6 Months Before Intervention (Oct 2015–Mar 2016) No. (%)	First 6 Months of Intervention (Apr 2016–Sep 2016) No. (%)	Second 6 Months of Intervention (Oct 2016–Mar 2017) No. (%)	Total No.
Pills^a^	2303 (10.8)	3378 (8.4)	3216 (7.6)	8897
DMPA-IM	4292 (20.0)	9115 (22.7)	11,933 (28.3)	25,340
DMPA-SC	—	6470 (16.1)	7803 (18.5)	14,273
Implants	1926 (9.0)	2140 (5.3)	2262 (5.4)	6328
IUD	217 (1.0)	280 (0.7)	254 (0.6)	751
Permanent methods^b^	21 (0.1)	94 (0.2)	123 (0.3)	238
Condoms^c^	12,662 (59.1)	18,646 (46.5)	16,540 (39.3)	47,848
**Total**	**21,421 (100.0)**	**40,123 (100.0)**	**42,131 (100.0)**	**103,675**
**Total CYPs**	**7073**	**11,491**	**13,009**	**31,573**

Abbreviations: CYP, couple-years of protection; DMPA-IM, intramuscular depot medroxyprogesterone acetate; DMPA-SC, subcutaneous depot medroxyprogesterone acetate; IUD, intrauterine device.

a Includes combined oral contraceptives and progesterone-only pills.

b Includes tubal ligation and vasectomy.

c Includes both male and female condoms.

### Injectables' Share of the Family Planning Market

The increase in the share of injectables (DMPA-IM and DMPA-SC) was far greater than for other methods. The volume of injectables provided increased from 4,292 units during the 6 months before the project began (DMPA-IM only), to 15,585 units during the first 6 months of the intervention, and further to 19,736 during the second 6 months of project implementation (DMPA-IM and DMPA-SC combined during project implementation). As a result, there were notable changes in the mix of family planning methods provided across project sites, with injectables growing to represent a significantly larger proportion of methods provided during the first and second 6 months of project implementation (43%) than before implementation (20%) (Table 4).

### Methods Provided by Service Delivery Channel

**FIGURE 2.**
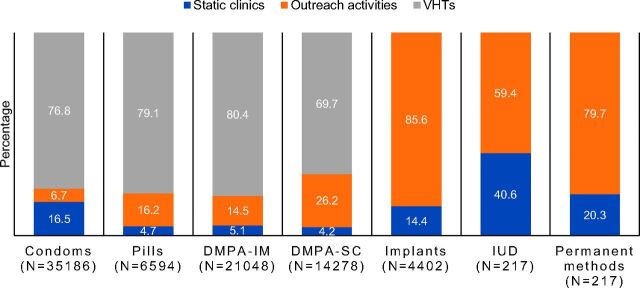
Percentage Distribution of Family Planning Methods by Service Delivery Channel, 4 Districts of Uganda, April 2016–March 2017 Abbreviations: DMPA-IM, intramuscular depot medroxyprogesterone acetate; DMPA-SC, subcutaneous depot medroxyprogesterone acetate; IUD, intrauterine device; VHT, village health team. Note: Pills include combined oral contraceptives and progestin-only pills; condoms include male and female condoms; and permanent methods include tubal ligation and vasectomy.

The vast majority of all short-acting family planning methods provided through the project were delivered at the community level, either by VHTs or through mobile outreach clinics (Figure 2). The majority of implants (86%) and IUD insertions (59%) were also accessed in the community, at mobile outreach sites. DMPA-SC services were overwhelmingly provided at the community level by VHTs (70%) or through mobile outreach services (26%).

**Figure:**
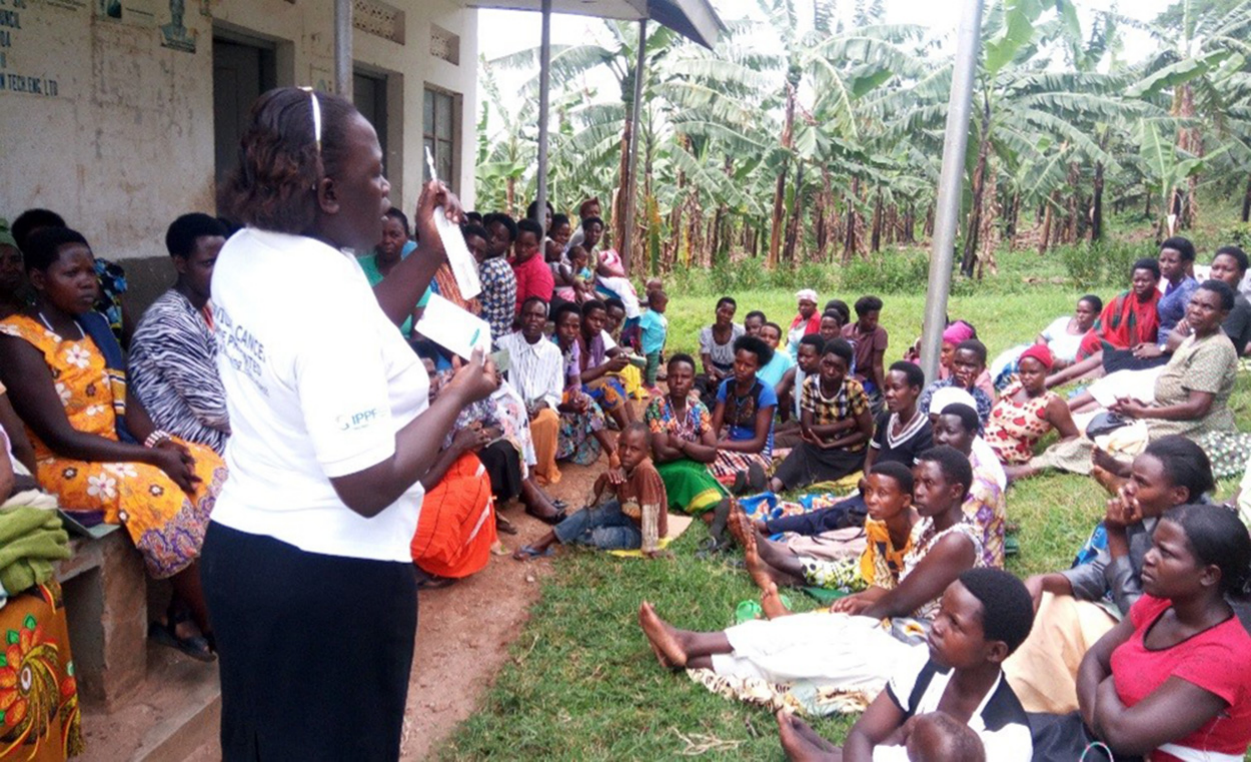
During an RHU outreach event in Kabarole district, Uganda, a provider shows women waiting for family planning services what Sayana Press looks like and provides information about the method. © 2016 Irene Kugonza/RHU.

### Age Profile of Clients by Family Planning Method

Overall, 42% of family planning services provided through the project across all project sites were delivered to young people (aged below 25). However, there were significant variances between methods. Notably, a significantly higher proportion of DMPA-SC units (43%) were administered to young people than units of DMPA-IM (37%), oral contraceptives (38%), implants (22%), or IUDs (17%) (Table 5). Further analysis of DMPA-SC services by age and service delivery channel revealed that VHTs served a higher proportion (45%) of young DMPA-SC clients than static clinics (35%) or mobile outreach (36%) (results not shown).

A significantly higher proportion of DMPA-SC units were administered to young women compared with other methods, except condoms.

**TABLE 5.** Percentage Distribution of Family Planning Clients by Age and Method, 4 Districts of Uganda, April 2016–March 2017

Method	Age Category		Total (N)
	<25 Years (%)	≥25 Years (%)	
Pills	38.4	61.6	6594
DMPA-IM	36.8	63.2	21,048
DMPA-SC	42.6	57.4	14,273
Implants	21.6	78.4	4402
IUD	16.9	83.1	534
Permanent methods	2.3	97.7	217
Condoms	48.1	51.9	35,186
**All methods**	**42.1**	**57.9**	**82,254**

Abbreviations: DMPA-IM, intramuscular depot medroxyprogesterone acetate; DMPA-SC, subcutaneous depot medroxyprogesterone acetate; IUD, intrauterine device.

Note: Pills include combined oral contraceptives and progestin-only pills; condoms include male and female condoms; and permanent methods include tubal ligation and vasectomy.

### Enabling Factors for the Provision of DMPA-SC at the Community Level

From qualitative interviews, we identified several factors that enabled introduction of DMPA-SC.

#### Training and Supportive Supervision

Facility- and community-based providers recalled receiving comprehensive training on DMPA-SC and also responded positively to training on counseling skills for other methods as part of promoting voluntary family planning services.

*We were taught comprehensive family planning methods including Sayana Press. About Sayana Press, we were taught about the size of injection, its strength, its user-friendliness compared to other injectables, storage, side effects, how it works. … I think we covered a comprehensive package of Sayana Press.* (Male facility-based provider, 10 years' experience)

However, some providers felt that refresher training on DMPA-SC would add value and enable them to solidify their learning.

*The training was adequate; however, they should have organized another training so that they can refresh us properly. As a people, we may be forgetful or there may be something that we have not done the right way. So, if they come and organize a refresher meeting then you can correct your mistakes.* (Female, VHT, 17 years' experience)

Most VHTs considered supervision important for strengthening their skills, improving quality of services, and building confidence among community members.

*Yes, the in-charge of [facilities] is supporting us so much. When he comes to the ground [with other staff] for outreaches, people get to know that I am not doing it alone as an individual. So, we are supported. When we are trained in class, we assume we understand everything, but he comes and reminds you of how to prepare the drugs for administration.* (Female, VHT, 10 years' experience)

#### Reliable Commodity Supplies and Linkages

A reliable supply of family planning commodities was considered essential for providing DMPA-SC and other family planning services. Most VHTs indicated that there were no challenges with DMPA-SC stock-outs and other materials or supplies necessary for its provision during project implementation.

*From the time [DMPA-SC] was introduced, it has never been out of stock. I usually come here [RHU clinic] to request for it and it is provided, so there has not been any problem.* (Female, VHT, 22 years' experience)

The strong relation between VHTs and facilities was considered important for enabling commodity resupply and referral of clients for long-acting methods.

*First of all, it's a teamwork; I always work well with [health care providers] at the health center. We understand each other and also I see there is a lot of cooperation because they always call me and give me assignments and I am always willing to help.* (Male, VHT, 7 years' experience)

#### Community Acceptance

Early engagement with, and sensitization of, community stakeholders was identified as beneficial to the smooth introduction of DMPA-SC.

*I know about Sayana Press and we attended the district entry meeting where SP [Sayana Press] was introduced to the district. Because this is a new method, we needed to be aware. The orientation meeting gave us much information and knowledge.* (Key informant interviewee, District Health Officer)

#### Providers' View on Workload

Many providers felt that DMPA-SC expanded the range of available family planning methods without significantly affecting their workload. DMPA-SC is similar to DMPA-IM, which is already being provided by active VHTs in their communities. Some facility-based providers reported that because DMPA-SC is easier to administer than DMPA-IM and implants, its introduction had actually enabled them to serve more clients in less time.

Community health workers were positive about the addition of DMPA-SC service delivery to their responsibilities.

*Family planning has long been there, and we have been providing family planning services to people, so Sayana Press is not any different. It is not complicated to administer. It is normal with us and we give it to people who want it easily. It is not any different from any other family planning method.* (Female VHT, 10 years' experience)

*It has reduced the workload because it is very easy to give. … It is very easy to give compared to Depo Provera and these other methods like Implanon. It has reduced our workload.* (Female facility-based provider, 4 years' experience)

### Barriers to the Introduction of DMPA-SC

Interviews with the key informants also identified a range of inhibiting factors.

#### Inadequate Number of Trained Service Providers

Some key informants indicated that the number of community-based health workers trained to provide injectable contraceptives was still inadequate to meet the rising demand.

*Only a few of us were trained. Some VHTs in some sub-counties were not trained in family planning services, so we cover bigger areas and sometimes we are not able to reach some areas. Hence, people in those areas miss out on those services. It is important to increase the number of VHTs to publicize family planning services.* (Female, VHT, 5 years' experience)

#### Lack of Transportation

High demand, large catchment areas, and poor transport connections left VHTs feeling stretched.

*I am skilled and comfortable providing it [DMPA-SC] to people who need it. I also want to provide it to other people in far places only if I get [transport] facilitation. This acts as a barrier to me. … lack of transport facilitation to reach women in the far areas.* (Female, VHT, 10 years' experience)

## DISCUSSION

This study documents the experience of an NGO in introducing DMPA-SC at the facility and community levels in 4 districts of Uganda. A number of themes emerge from the quantitative and qualitative data collected through the project.

### The Viability of Community-Based Distribution of DMPA-SC

More than half of all methods, and 96% of DMPA-SC units, provided through the project were delivered at the community level outside of static clinics—either by VHTs or through mobile outreach teams. On average, each VHT provided 21 units of DMPA-SC per month, equating to an estimated 62 CYPs and 22 unintended pregnancies averted per VHT, per year. In the absence of service statistics from public health facilities, we do not have a complete picture of where clients accessed family planning services across all project districts. However, RHU's experience demonstrates that strengthening VHTs' capacity through training and ensuring continuous supply of contraceptive commodities can result in the provision of a high volume of family planning services at the community level.

96% of DMPA-SC units provided through the project were delivered at the community level.

### Potential for DMPA-SC Introduction to Increase the Share of Injectables in the Ugandan Method Mix

Project data showed that there was a shift in the mix of family planning services provided during the project period, with injectables growing to represent 43% of all methods provided compared with just 20% prior to implementation. Project-level service data from a small number of service outlets (4 RHU static clinics, 40 VHTs, and 26 outreach sites) cannot be interpreted in the same way as population-based sample data such as from the Demographic and Health Surveys. However, the observed shift in the comparative balance of methods provided at the service level does provide insight into how the introduction of DMPA-SC could potentially further increase the share of injectables within the method mix in Uganda once it is rolled out nationally. Injectable contraceptives have become the most commonly used modern methods in a number of sub-Saharan African countries.^18^ The use of injectables has risen to about 15% to 20% of married women, equaling about 40% of all contraceptive use, with this percentage even higher in some countries.^19^^–^^21^

The share of injectables in the method mix grew from 20% at baseline to 43% at the end of the project.

Importantly, while the introduction of DMPA-SC has the potential to impact the relative share of injectables within the method mix, this would not necessarily equate to a decline in the uptake of other methods in absolute numbers. Within the RHU project, for example, there was a significant increase in the volume of *all* contraceptive methods provided at project sites including long-acting methods. Furthermore, there is evidence to suggest that DMPA-SC has the potential to reach new acceptors of family planning.^8^ However, more rigorous research is needed on the possible impact of DMPA-SC introduction on the uptake of other methods.

It is important that, as with any new method, programmers use the introduction of DMPA-SC as an opportunity to reinforce access to a full range of methods. For example, programmers may consider giving community- and facility-based health workers refresher training on *all methods* when they are introducing DMPA-SC; strengthening links between service delivery points to enable referral for long-acting methods; and ensuring continuous availability of all contraceptive commodities across the family planning delivery system. These approaches, adopted by RHU in this project, were widely welcomed and identified through stakeholder interviews as enabling factors to the introduction of DMPA-SC.

### The Apparent Popularity of DMPA-SC Among Young People

Project data on the proportion of family planning services provided by client age group reinforces existing evidence that DMPA-SC is attractive to young people.^8^^,^^22^ Nearly half (43%) of all DMPA-SC units administered were provided to clients below 25 years of age—a significantly higher proportion than for all methods, except condoms. Other studies have found that DMPA-SC is popular among young people because of its convenience, the potential for discreet use without partner/parental detection, and the lower reported side effects than with some other methods.^13^^,^^23^^,^^24^ Service data show that a greater proportion of VHT clients were young (47%) than static clinic clients (35%) or mobile outreach clients (36%). This could suggest that it is easier or more comfortable for young people to access contraceptives via VHTs, away from formal health settings.

Our pilot results reinforce that DMPA-SC is attractive to young people.

### Both Supply- and Demand-Side Factors Facilitated Provision of DMPA-SC Services

From qualitative interviews, we identified several factors that enabled introduction of DMPA-SC at the community level that may be of note for programmers and policy makers. VHTs and facility-based providers valued the comprehensive family planning training; consistent availability of contraceptive commodities; and strong referral links (between public and NGO providers, and from VHTs to static facilities). Key informant interviews also found that engagement of community leaders before service delivery began was an important enabling factor.

However, a number of barriers and challenges remain. VHTs reported that the transport allowances they received through this project were insufficient to cover the cost of traveling to visit clients and to restock commodities at static clinics. There is need to review VHT support packages to better enable them to effectively do their work. In addition, the project trained only a limited number of active VHTs (10 in each district). Inadequate number of trained VHTs limited provision of DMPA-SC in the community. A 2015 study estimated that there were 179,175 VHTs in Uganda—30% of whom did not have basic training at that time.^25^ As national rollout plans for DMPA-SC progress, training VHTs, and training them *at volume*, will likely become a major priority and challenge for government and partners alike.

### Study Limitations

The project provided significant support in the form of training to the public sector to enable comprehensive service delivery in static facilities. However, due to the absence of data from public-sector facilities, the data presented do not offer a full picture of the intervention. Analysis is therefore limited to the volume of methods provided at RHU clinics, through mobile outreach, and by VHTs.

The absence of unique client identifiers across all service delivery points also limits analysis in several ways. First, it was not possible to identify how many individual clients were served by the project, and we should expect that a proportion of clients were provided with a method on multiple occasions during the course of the project, particularly users of short-acting methods including DMPA-SC. The absence of unique client identifiers across some service delivery platforms also means that it was not possible to track switching between methods.

Interviewees were purposively identified based on their role in the intervention and could have been tempted to offer positive views of the program. Given that some participants reported challenges, however, the selection process did not appear to bias the qualitative data collection to only those with positive views.

## CONCLUSIONS

As questions about safety, efficacy, and acceptability of DMPA-SC are satisfactorily answered through rigorous research,^26^^–^^28^ programmers and policy makers turn their attention to operational questions about how DMPA-SC can be rolled out effectively to increase access to and reach those most in need. RHU's experience gives insight into one NGO's experience in introducing DMPA-SC at the community level in non-trial settings. The project focused heavily on community distribution through VHTs, which appears to have been an effective strategy for increasing service delivery and reaching young people. The views of project stakeholders in Uganda offer insight into how program teams, nationally and internationally, can roll out DMPA-SC to effectively reach those in most need of voluntary family planning.

## Notes

**Peer Reviewed**

**Funding:** The project, also known as the Sayana Press Learning Project (SPLP), was funded by the United States Agency for International Development (USAID) through the Sustainable Networks Project (SIFPO2) and implemented by Reproductive Health Uganda (RHU) in collaboration with the Ugandan Ministry of Health (MOH) from April 2016 to March 2017.

**First Published Online:** November 14, 2018
