# Erratum for: Odwe et al., Introduction of Subcutaneous Depot Medroxyprogesterone Acetate (DMPA-SC) Injectable Contraception at Facility and Community Levels: Pilot Results From 4 Districts of Uganda

**DOI:** 10.9745/GHSP-D-19-00036

**Published:** 2019-03-22

See updated article.

In the article “Introduction of Subcutaneous Depot Medroxyprogesterone Acetate (DMPA-SC) Injectable Contraception at Facility and Community Levels” by George Odwe and colleagues, which appeared in the December 2018 issue (Volume 6, Number 4), in Table 4, the content for footnotes ‘b’ and ‘c’ were reversed. This has now been corrected.

